# Independent component analysis: recent advances

**DOI:** 10.1098/rsta.2011.0534

**Published:** 2013-02-13

**Authors:** Aapo Hyvärinen

**Affiliations:** Department of Computer Science, Department of Mathematics and Statistics, and HIIT, University of Helsinki, Helsinki, Finland

**Keywords:** independent component analysis, blind source separation, non-Gaussianity, causal analysis

## Abstract

Independent component analysis is a probabilistic method for learning a linear transform of a random vector. The goal is to find components that are maximally independent and non-Gaussian (non-normal). Its fundamental difference to classical multi-variate statistical methods is in the assumption of non-Gaussianity, which enables the identification of original, underlying components, in contrast to classical methods. The basic theory of independent component analysis was mainly developed in the 1990s and summarized, for example, in our monograph in 2001. Here, we provide an overview of some recent developments in the theory since the year 2000. The main topics are: analysis of causal relations, testing independent components, analysing multiple datasets (three-way data), modelling dependencies between the components and improved methods for estimating the basic model.

## 1. Introduction

It is often the case that the measurements provided by a scientific device contain interesting phenomena mixed up. For example, an electrode placed on the scalp as in electroencephalography measures a weighted sum of the electrical activities of many brain areas. A microphone measures sounds coming from different sources in the environment. On a more abstract level, a gene expression level may be considered the sum of many different biological processes. A fundamental goal in scientific enquiry is to find the underlying, original signals or processes that usually provide important information that cannot be directly or clearly seen in the observed signals.

Independent component analysis (ICA; Jutten & Hérault [1])  has  been  established as a fundamental way of analysing such multi-variate data. It learns a linear decomposition (transform) of the data, such as the more classical methods of factor analysis and principal component analysis (PCA). However, ICA is able to find the underlying components and sources mixed in the observed data in many cases where the classical methods fail.

ICA attempts to find the original components or sources by some simple assumptions of their statistical properties. Not unlike in other methods, the underlying processes are assumed to be independent of each other, which is realistic if they correspond to distinct physical processes. However, what distinguishes ICA from PCA and factor analysis is that it uses the non-Gaussian structure of the data, which is crucial for recovering the underlying components that created the data.

ICA is an unsupervised method in the sense that it takes the input data in the form of a single data matrix. It is not necessary to know the desired ‘output’ of the system, or to divide the measurements into different conditions. This is in strong contrast to classical scientific methods based on some experimentally manipulated variables, as formalized in regression or classification methods. ICA is thus an exploratory, or data-driven method: we can simply measure some system or phenomenon without designing different experimental conditions. ICA can be used to investigate the structure of the data when suitable hypotheses are not available, or they are considered too constrained or simplistic.

Previously, we wrote a tutorial on ICA [2] as well as a monograph [3]. However, that material is more than 10 years old, so our purpose here is to provide an update on some of the main developments in the fields since the year 2000 (see Comon & Jutten [4] for a recent in-depth reference). The main topics we consider below are:

— causal analysis, or structural equation modelling (SEM), using ICA (§3);
— testing of independent components for statistical significance (§4);
— group ICA, i.e. ICA on three-way data (§5);
— modelling dependencies between components (§6); and
— improvements in estimating the basic linear mixing model, including ICA using time–frequency decompositions, ICA using non-negative constraints, and modelling component distributions (§7).

We start with a very short exposition of the basic theory in §2.

## 2. Basic theory of independent component analysis

In this section, we provide a succinct exposition of the basic theory of ICA before going to recent developments in subsequent sections.

### (a) Definition

**Figure 1.**
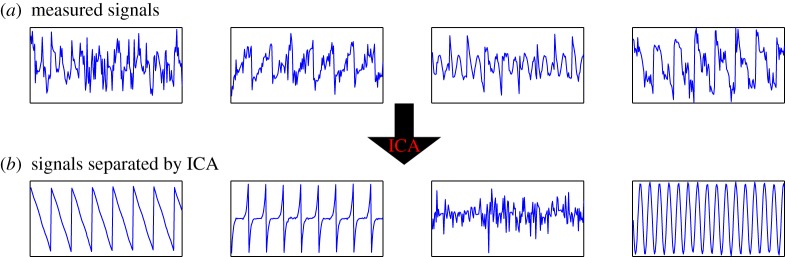
The basic idea of ICA. From the four measured signals shown in (*a*), ICA is able to recover the original source signals that were mixed together in the measurements, as shown in (*b*). (Online version in colour.)

Let us denote the observed variables by *x*_*i*_(*t*), *i*=1,…,*n*, *t*=1,…,*T*. Here, *i* is the index of the observed data variable and *t* is the time index, or some other index of the different observations. The *x*_*i*_(*t*) are typically signals measured by a scientific device. We assume that they can be modelled as linear combinations of hidden (latent) variables *s*_*j*_(*t*),*j*=1,…,*m*, with some unknown coefficients *a*_*ij*_,
2.1
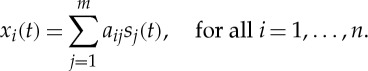
The fundamental point is that we observe only the variables *x*_*i*_(*t*), whereas both *a*_*ij*_ and *s*_*i*_(*t*) are to be estimated or inferred. The *s*_*i*_ are the independent components, whereas the coefficients *a*_*ij*_ are called the mixing coefficients. This estimation problem is also called blind source separation. The basic idea is illustrated in figure 1.

The model can be expressed in different ways. Typically, the literature uses the formalism where the index *t* is dropped, and the *x*_*i*_ and the *s*_*i*_ are considered random variables. Furthermore, the *x*_*i*_ are usually collected into a vector **x** of dimension *n*, the same is done for the *s*_*i*_ and the coefficients *a*_*ij*_ are collected into a mixing matrix **A** of size *n*×*n*. (In this paper, vectors are denoted by bolded lowercase letters and matrices are bolded uppercase. Random variables and their realizations are not typographically different, but the index *t* always denotes realizations.) Then, the model becomes
2.2
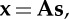
where **x** and **s** are now random vectors, and **A** is a matrix of parameters. We can equally well move to a matrix notation where the observed *x*_*i*_(*t*) are collected into a *n*×*T* matrix **X**, with *i* giving the row index and *t* giving the column index, and likewise for *s*_*i*_(*t*), giving
2.3
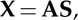
where **A** is still the same matrix as in (2.2). A proper probabilistic treatment really requires the formulation in (2.2) because in that formalism, we have random variables as in typical statistical theory, and we can talk about their expectations, in particular, in the limit of an infinite number of observations. The formulation in (2.3) is not suitable for probabilistic treatment in the same way because the matrix **X** is now fixed by the observations and not random; however, it is useful in other ways, as will be seen in the following.

### (b) Identifiability

The main breakthrough in the theory of ICA was the realization that the model can be made identifiable by making the unconventional assumption of the non-Gaussianity of the independent components [5]. More precisely, assume the following.

— The components *s*_*i*_ are mutually statistically independent. In other words, their joint density function is factorizable: 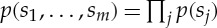.
— The components *s*_*i*_ have non-Gaussian (non-normal) distributions.
— The mixing matrix **A** is square (i.e. *n*=*m*) and invertible.

Under these three conditions, the model is essentially identifiable [5,6]. This means that the mixing matrix and the components can be estimated up to the following rather trivial indeterminacies: (i) the signs and scales of the components are not determined, i.e. each component is estimated only up to a multiplying scalar factor, and (ii) any ordering of the components is not determined.

The assumption of independence can be seen as a rather natural ‘default’ assumption when we do not want to postulate any specific dependencies between the components. It is also more or less implicit in the theory of classical factor analysis, where the components or factors are assumed uncorrelated and Gaussian, which implies that they are independent (more on this below). A physical interpretation of independence is also sometimes possible: if the components are created by physically separate and non-interacting entities, then they can be considered statistically independent.

On the other hand, the third assumption is not necessary and can be relaxed in different ways, but most of the theory makes this rather strict assumption for simplicity.

So, the real fundamental departure from conventional multi-variate statistics is to assume that the components are non-Gaussian. Non-Gaussianity also gives a new meaning to independence: for variables with a joint Gaussian distribution, uncorrelatedness and independence are in fact equivalent. Only in the non-Gaussian case is independence something more than uncorrelatedness. Uncorrelatedness is assumed in other methods such as PCA and factor analysis, but this non-Gaussian form of independence is usually not.

As a trivial example, consider two-dimensional data that are concentrated on four points: (−1,0),(1,0),(0,−1),(0,1) with equal probability 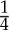. The variables *x*_1_ and *x*_2_ are uncorrelated owing to symmetry with respect to the axes: if you flip the sign of *x*_1_, the distribution stays the same, and thus we must have *E*{*x*_1_*x*_2_}=*E*{(−*x*_1_)*x*_2_}, which implies their correlation (and covariance) must be zero. On the other hand, the variables clearly are not independent because if *x*_1_ takes the value −1, we know that *x*_2_ must be zero.

### (c) Objective functions and algorithms

Most ICA algorithms divide the estimation of the model into two steps: a preliminary whitening and the actual ICA estimation. Whitening means that the data are first linearly transformed by a matrix **V** such that **Z**=**VX** is white, i.e.
2.4
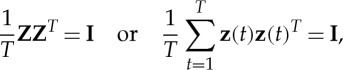
where **I** is the identity matrix. Such a matrix **V** can be easily found by PCA: normalizing the principal components to unit variance is one way of whitening data (but not the only one).

The utility of this two-step procedure is that after whitening, the ICA model still holds,
2.5
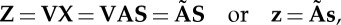
where the transformed mixing matrix 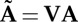 is now *orthogonal* [5,2]. Thus, after whitening, we can constrain the estimation of the mixing matrix to the space of orthogonal matrices, which reduces the number of free parameters in the model. Numerical optimization in the space of orthogonal matrices tends to be faster and more stable than in the general space of matrices, which is probably the main reason for making this transformation.

It is important to point out that whitening is not uniquely defined. In fact, if **z** is white, then any orthogonal transform **Uz**, with **U** being an orthogonal matrix, is white as well. This highlights the importance of non-Gaussianity: mere information of uncorrelatedness does not lead to a unique decomposition. Because, for Gaussian variables, uncorrelatedness implies independence, whitening exhausts all the dependence information in the data, and we can estimate the mixing matrix only up to an arbitrary orthogonal matrix. For non-Gaussian variables, on the other hand, whitening does not at all imply independence, and there is much more information in the data than what is used in whitening.

For whitened data, considering an orthogonal mixing matrix, we estimate 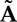 by maximizing some objective function that is related to a measure of non-Gaussianity of the components. For a tutorial treatment on the theory of objective functions in ICA, we refer the reader to Hyvärinen & Oja [2] and Hyvärinen *et al.* [3]. Basically, the main approaches are maximum-likelihood estimation [7], and minimization of the mutual information between estimated components [5]. Mutual information is an information-theoretically motivated measure of dependence; so its minimization is simply motivated by the goal of finding components that are as independent as possible. Interestingly, both of these approaches lead to essentially the same objective function. Furthermore, a neural network approach called infomax was proposed by Bell & Sejnowski [8] and Nadal & Parga [9], and was shown to be equivalent to likelihood by Cardoso [10].

The ensuing objective function is usually formulated in terms of the inverse of 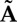, whose rows are denoted by 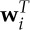, as
2.6
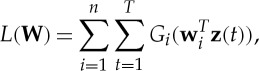
where *G*_*i*_ is the logarithm of the probability density function (pdf) of *s*_*i*_, or its estimate 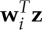. In practice, quite rough approximations of the log-pdf are used; the choice 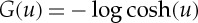, which is essentially a smoothed version of the negative absolute value function −|*u*|, works well in many applications. This function is to be maximized under the constraint of orthogonality of the **w**_*i*_. The **z**(*t*) are here the observed data points that have been whitened.

Interestingly, this objective function depends only on the marginal densities of the estimated independent components 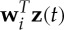. This is quite advantageous because it means we do not need to estimate any dependencies between the components, which would be computationally very complicated.

Another interesting feature of the objective function in (2.6) is that each term 
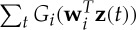
 can be interpreted as a measure of non-Gaussianity of the estimated component 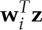. In fact, this is an estimate of the negative differential entropy of the components, and differential entropy can be shown to be maximized for a Gaussian variable (for fixed variance). Thus, ICA estimation is essentially performed by finding uncorrelated components that maximize non-Gaussianity (see Hyvärinen & Oja [2] and Hyvärinen *et al.* [3] for more details).

Such objective functions are then optimized by a suitable optimization method, the most popular ones being FastICA [11] and natural gradient methods [12].

## 3. Causal analysis, or structural equation modelling

We start the review of recent developments by considering a rather unexpected application of the theory of ICA found in causal analysis. Consider the following fundamental question: the observed random variables *x*_1_ and *x*_2_ are correlated, and we want to know which one causes which. Is *x*_1_ the cause and *x*_2_ the effect, or vice versa? In general, such a question cannot be answered, and the answer could also be ‘neither’ or ‘both’ of them causing the other. However, we can make some progress in this extremely important question by postulating that one of the variables has to be the cause and the other one the effect.

If we further assume that the connection between the two variables takes the form of a linear regression model, we are basically left with the following model selection problem. Choose between the following two models:
3.1
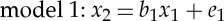
and
3.2
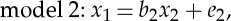
where *b*_1_ and *b*_2_ are regression coefficients. Now, if model 1 holds, we can say that *x*_1_ causes *x*_2_, and if model 2 holds, we can say that *x*_2_ causes *x*_1_. The residuals *e*_1_,*e*_2_ are assumed to be independent of the regressors *x*_1_ and *x*_2_, respectively.

The classical problem with such model selection is that it is not possible for Gaussian variables. If we assume the data are Gaussian, the two models give equally good fits. In fact, if we assume the variables *x*_1_ and *x*_2_ are standardized to unit variance, the regression coefficients are equal, i.e. *b*_1_=*b*_2_; they are equal to the correlation coefficient between *x*_1_ and *x*_2_. The variances of the residuals are thus also equal, and the models are completely symmetric with respect to *x*_1_ and *x*_2_. There is no way of distinguishing between the two models.

However, if the data are non-Gaussian, the situation is different. We can formulate the two models as ICA models,
3.3
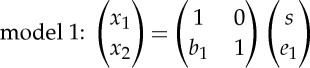
and
3.4
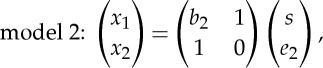
where one of the components turns out to be equal to one of the observed variables. The two components on the right-hand side are, by definition, independent and non-Gaussian; so these are proper ICA models. Thus, selecting the direction of causality is simply reduced to choosing between two ICA models.

In principle, we could just estimate ICA on the vector **x**=(*x*_1_,*x*_2_) and see whether the mixing matrix is closer to the form of the one in model 1 or model 2. The zeros in the mixing matrices are in different places, which clearly distinguish them. A more efficient way of choosing between the models can be based on likelihood ratios of the two models [13,14]. (An earlier approach used cumulants [15].)

In fact, this is just a special case of the general problem of estimating a linear Bayesian network, or an SEM. In the general SEM, we model the observed data vector **x** as
3.5
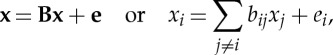
with a matrix **B** that has zeros in the diagonal. The idea that each *x*_*i*_ is a function of the other *x*_*j*_ formalizes the causal connections between the different variables. The theory of SEM has a long history, but most of it is based on Gaussian models, and leads to the same kind of identifiability problems as estimation of the basic linear mixing model (2.2) with Gaussian variables.

The linear non-Gaussian acyclic model (LiNGAM) was introduced by Shimizu *et al.* [16] as a general framework for causal analysis based on estimation of (3.5). The assumption of non-Gaussianity of the *e*_*i*_ is combined with the assumption of acyclicity to yield perfect identifiability of the model. The assumption of acyclicity is quite typical in the theory of Bayesian networks: it means that the graph describing the causal relations (defined by the matrix **B**) is not allowed to have cycles. Thus, the directions of causality are always well defined: if *x*_*i*_ causes *x*_*j*_, then it is not possible that *x*_*j*_ causes *x*_*i*_, even indirectly. However, such acyclicity can be relaxed [17,14].

**Figure 2.**
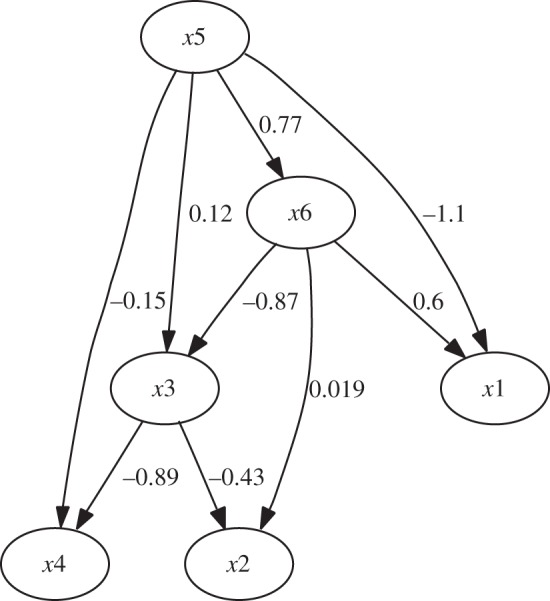
An example of a causal network between the variables *x*_*i*_ that can be estimated with LiNGAM. The non-zero *b*_*ij*_'s are shown as arrows from *x*_*j*_ to *x*_*i*_, with numerical values attached to them. The network is acyclic, which is seen in the fact that after a suitable reordering of the variables (which has been done here), all the arrows go down.

An example of a network that can be estimated by LiNGAM is shown in figure 2.

The simplest method of estimating the LiNGAM model is to first perform ICA on the data, and then infer the network structure, i.e. the matrix **B** from the mixing matrix of ICA. In principle, this may seem straightforward because (3.5) implies **x**=(**I**−**B**)^−1^**e**, and thus **B** is very closely related to the mixing matrix. However, the situation is much more complicated because ICA does not give the components in any specific order, whereas the SEM defines a specific order for the *e*_*i*_ in the sense that each *e*_*i*_ corresponds to *x*_*i*_ (and not *x*_*i*−1_, for example). Thus, more sophisticated methods are needed to infer the correct ordering, for example, based on acyclicity [16,18].

Estimating non-Gaussian Bayesian networks is a topic of intense research at the moment. Different extensions of the basic framework consider temporal structure [19], and three-way structure [20,21]. It is also possible to estimate nonlinear models, in which case non-Gaussianity may no longer be necessary [22,23]. As already mentioned, cyclic models can be estimated, replacing the acyclicity assumption by a weaker one [17,14].

## 4. Testing of independent components

After estimating ICA, it would be very useful to assess the reliability or statistical significance of the components. In fact, in the literature, independent components estimated from various kinds of scientific data are often reported without any kind of validation, which seems to be against the basic principles of scientific publication.

The classical validation of estimation results is statistical significance (also called reliability), which assesses if it is likely that the results could be obtained by chance. In the context of ICA, we would like to be able to say if a component could be obtained, for example, by inputting just pure noise to an ICA algorithm.

An additional problem that we encounter with computationally intensive and complex estimation methods is what we could call computational reliability. Even if the data were perfect and sufficient for any statistical inference, the computational algorithm may get stuck in bad local optima or otherwise fail to produce meaningful results. Most ICA algorithms are based on local optimization methods: they start from a random initial point and try to increase the objective function at every iteration. There is absolutely no guarantee that such an algorithm will find the real (global) optimum of the objective function. This is an additional source of randomness and errors in the results [24].

To validate ICA results, it might seem, at first sight, to be interesting to test the independence of the components because this is an important assumption in the model. In practice, however, this is not very relevant because ICA methods can often estimate the decomposition quite well, even if the components are far from independent, as discussed in §6 below. What is important in practice is to assess whether the components correspond to some real aspects of the data, regardless of the exact validity of the model assumptions.

One way to assess the reliability of the results is to perform some randomization of the data or the algorithm, and see whether the results change a lot [25,24]. To assess the statistical significance, we could randomize the data, for example, by bootstrapping. To assess computational reliability, we could run the ICA algorithm from many different initial points. An additional difficulty for such assessment in the case of ICA is the permutation indeterminacy: the components are given by the algorithm in a random order. Thus, we have to match the components from different runs.

The results of such randomization can be visualized by projecting the components from the high-dimensional component space onto, say, a two-dimensional plane [24]. If an almost identical component is output by the algorithm for all, or most of, the randomized runs, it is more likely to be a true phenomenon in the data and not a random result.

In addition to such visualization, recently developed methods allow the statistical quantification of the reliability of the components. Such a method seems to be difficult to obtain for bootstrapping; so it was proposed by Hyvärinen [26] that one should analyse a number of separate datasets. If the independent components are similar enough in the different datasets, one can assume that they correspond to something real. In some applications, one naturally obtains a number of data matrices that one would expect to contain the same independent components. In the case of neuroimaging, for example, one typically measures brain activities of many subjects, and tries to find components that the subjects have in common [27]. In general, even if one only measures a single dataset, one can just divide it into two or more parts.

Using this idea of analysing different datasets, it is actually possible to formulate a proper statistical testing procedure, based on a null hypothesis, which gives *p*-values for each component. The key idea is to consider the baseline where the orthogonal transformation 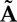 estimated after whitening is completely random; this gives the null distribution that models the chance level [26]. In the space of orthogonal matrices, it is in fact possible to define ‘complete randomness’ as the uniform distribution in the set of orthogonal matrices owing to the compactness of that set. To see whether a component is significantly similar in the different datasets, one computes the distribution of the similarities of the components under this null distribution and compares its quantiles with the similarities obtained for the real data. This gives a statistically rigorous method for assessing the reliability of the components. The similarities can be computed either between the mixing coefficients corresponding to each component [26] or between the actual values of the independent components [28], depending on the application.

## 5. Group independent component analysis, or three-way data

In some applications, one does not measure just a single data matrix but several, as already pointed out in §4. In other words, the random vector **x** is measured under different experimental conditions, for different subjects, simply in different measurement sessions, etc. This gives rise to what is called three-way or three-mode data, which is properly described by three indices, for example, *x*_*i*,*k*_(*t*) where *i* is the index of the measured variable, *t* is the time index or a similar sample index, and *k*=1,…,*r* is the new index of the subject, the experimental condition or some similar aspect that gives rise to several matrices.

This is often called the problem of group ICA because most of the literature on the topic has been developed in the context of neuroimaging, where the problem is to analyse a group of subjects [29]. In that context, *k* is the index of the subject.

There are basically two approaches to the group ICA problem. One is the approach already described in §4: We do ICA separately on each data matrix and then combine the results, which further gives us the opportunity to test the significance of the components. The second approach, which we consider in this section, is to estimate some ‘average’ decomposition. For example, if we assume that the mixing matrices are approximately the same, then we can try to estimate the average mixing matrix.

The first, fundamental question in analysis of such three-way data is whether the three-way structure can be simply transformed into an ordinary two-way structure without losing too much information. In other words, can we just ‘collapse’ the data into an ordinary matrix and analyse it with ICA, or do we need special methods? In fact, in many cases where ICA is applied, it does not seem to be necessary to construct special methods for analysis of three-way data: it seems to be enough to transform the data into a single matrix for a useful application of ICA.

Denote by **X**_*k*_ the data matrix obtained from the *k*th condition (or subject). Its rows are the different variables *i*, and the columns different observations *t*. Thus, each **X**_*k*_ is a matrix that we could input to an ICA algorithm, which would model it as **X**_*k*_=**A**_*k*_**S**_*k*_.

Fundamentally, we can construct two different ‘total’ data matrices that contain all the **X**_*k*_, i.e. all the three-way data. We can concatenate the **X**_*k*_ either column-wise or row-wise, obtaining, respectively, the matrices 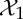 and 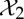,
5.1
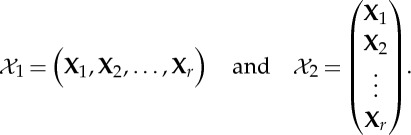
Which one we should use depends on what we expect to be similar over conditions/subjects *k*. If we assume that the mixing matrix is the same, but the components values are different, we should use 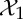 because we have
5.2
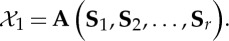
Thus, the ICA model holds for 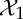, with the common mixing matrix **A**. Application of ordinary ICA on 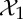 will estimate all the quantities involved.

By contrast, if we assume that the independent component matrices **S**_*k*_ are similar for the different subjects/conditions, while the mixing matrices are not, we should use 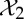 because we have
5.3
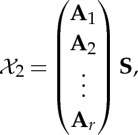
and thus the ICA model holds, with the common matrix of independent components **S**. Here, we can reduce the dimension of the data to *n*, the dimension of the original data matrices, and then perform ICA to obtain the common independent component matrix **S**. The mixing matrices **A**_*i*_ can be obtained afterwards, for example, by computing **A**_*k*_=**X**_*k*_**S**^*T*^/*T*. (A very interesting approach that further explicitly models (small) differences between the **S**_*k*_ was proposed by Varoquaux *et al.* [30].)

Doing ICA on 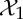 is typically quite straightforward. If the number of data points is computationally too large after concatenation, one can always take a smaller random sample of the columns of 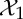 before inputting it into an ICA algorithm; this will have little effect on the results. On the other hand, 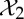 can have a very large dimension that can be quite problematic from a computational viewpoint. Different computational strategies are available to cope with this problem, as reviewed by Calhoun *et al.* [29]. A computationally efficient, if approximative, method was recently proposed by Hyvärinen & Smith [31].

If we can make even stronger assumptions on the similarities of the data matrices for different *k*, we can use methods developed for analysis of such three-way in the context of classical (Gaussian) multi-variate statistics. The most relevant method is parallel factor analysis or PARAFAC [32]. In the notation of ICA, the model assumed by PARAFAC can be expressed as
5.4
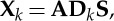
where **D**_*k*_ is a diagonal matrix, specific for each *k*. That is, the mixing matrices and independent components are the same for all *k* up to the scaling factors (and possibly switches of signs) given by **D**_*k*_. The differences between the conditions *k* are thus modelled by the diagonal matrices **D**_*k*_. PARAFAC is a major improvement to classical Gaussian factor analysis or PCA in the sense that it can actually uniquely estimate the components even for Gaussian data. However, there is an important restriction here, which is that the **D**_*k*_ must be linearly independent, which intuitively means that data matrices must be sufficiently different with respect to the scalings for different *k*. In fact, if all **D**_*k*_ were equal, the model would reduce an ordinary linear mixing like in (2.2).

A combination of ICA with PARAFAC for estimation of (5.4) was proposed by Beckmann & Smith [33], by basically assuming that the **S** in the PARAFAC model in (5.4) is non-Gaussian, like in ICA. This has the potential of improving estimation from what would be obtained by either ICA or PARAFAC alone. Estimation proceeds by considering the matrix 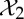, and maximizing an ICA objective function under some constraints on the mixing matrix. The constraints on the mixing matrix are a direct consequence of the definition of PARAFAC. On the other hand, if the data are non-Gaussian enough, under these assumptions, it might be enough to do ICA on the average data matrix 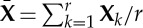 to estimate the average mixing matrix and the average components.

Three-way structure is related to a powerful approach to ICA based on joint diagonalization of covariance matrices. The idea is to estimate a number of covariance matrices, for example, in a number of time blocks, or in different frequency bands (which is related to estimating cross-correlation matrices with lags). Under suitable assumptions, joint (approximate) diagonalization of such covariance matrices estimates the ICA model, and a number of algorithms have been developed for such joint diagonalization [34,35,36]. Thus, these methods rely on an ‘artificial’ creation of three-way data from an ordinary data matrix. This suggests that when one actually has directly measured three-way data, such joint diagonalization approaches might be directly applicable and useful. A generalization of ICA based on this idea was proposed by Cardoso *et al.* [37].

## 6. Modelling dependencies of components

### (a) Why estimated components can be dependent

Often, the components estimated from data by an ICA algorithm are not independent. While the components are assumed to be independent in the model, the model does not have enough parameters to actually make the components independent for any given random vector **x**. This is because statistical independence is a very strong property with potentially an infinite number of degrees of freedom. In fact, independence of two random variables *s*_1_ and *s*_2_ is equivalent to any nonlinear transformations being uncorrelated, i.e.
6.1
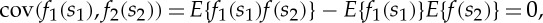
for *any* nonlinear functions *f*_1_ and *f*_2_, with *E*{.} denoting mathematical expectation. This is in stark contrast to uncorrelatedness, which means that (6.1) holds for the identity function *f*_1_(*s*)=*f*_2_(*s*)=*s*. This equation suggests that to find a transformation that is guaranteed to give independent components, we need an infinite number of parameters, i.e. a class of rather arbitrary nonlinear transformations. It is thus not surprising that linear transforms cannot achieve independence in the general case, i.e. for data with an arbitrary distribution. (See Hyvärinen *et al*. [38], ch. 9 for more discussion.)

In fact, if we consider a real dataset, it seems quite idealistic to assume that it could be a linear superposition of strictly independent components. A more realistic attitude is to assume that the components are bound to have some dependencies. Then, the central question is whether independence is a useful assumption for a particular dataset in the sense that it allows for estimation of meaningful components. In fact, empirical results tend to show that ICA estimation seems to be rather robust against some violations of the independence assumption.

On the other hand, modelling dependencies of the estimated components is an important extension of the analysis provided by ICA. It can give useful information on the interactions between the components or sources recovered by ICA. Thus, the fact that the components are dependent can be a great opportunity for gaining further insights into the structure of the data.

### (b) Correlation of squares of components

**Figure 3.**
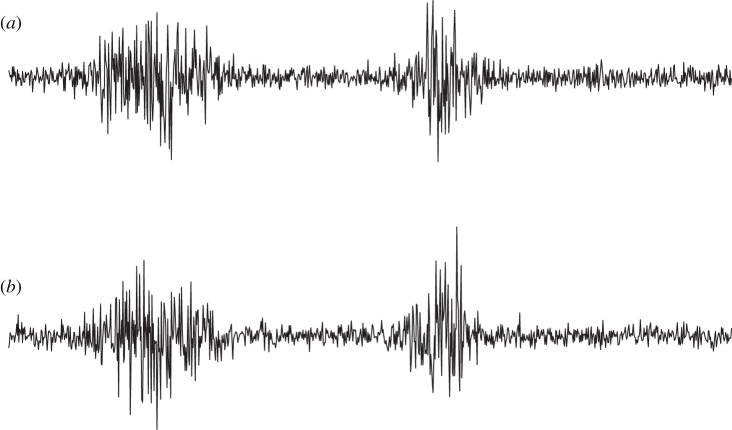
(*a*,*b*) An illustration of two signals whose activity levels are correlated, which leads to a correlation of their squares 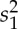 and 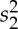. However, the signals are uncorrelated in the conventional sense.

A typical form of dependence in real data is correlation of variances or squares (also called correlation of energies owing to an abstract physics analogy). This typically means that there is some underlying process that determines the level of activity of the components, and the levels of activity are dependent of each other. An illustration of such signals is shown in figure 3.

The simplest way of modelling this process is to assume that the components are generated in two steps. First, a number of non-negative variance or scale variables *v*_*i*_ are created. These should be dependent on each other. Then, for each component, a zero-mean ‘original’ component 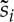 is generated independently of each other, and independently of the *v*_*i*_. Finally, the actual components *s*_*i*_ in the linear model (2.2) are generated as the products,
6.2
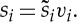
This generative model implies that the *s*_*i*_ are uncorrelated, but there is the correlation of squares [38],
6.3
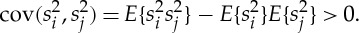


The extensions of ICA with correlations of squares essentially differ in what kind of dependencies they assume for the variance variables *v*_*i*_. In the earliest work, the *v*_*i*_ were divided into groups (or subspaces) such that the variables in the same group are positively correlated, while the variables in different groups are independent [39]. A follow-up paper made this division smooth so that the dependencies follow a ‘topographic’ arrangement on a two-dimensional grid, which allows for easy visualization and has interesting neuroscientific interpretations [40]. A fixed-point algorithm for the subspace model was proposed by Hyvärinen & Köster [41].

In those early models, the dependency structure of the *v*_*i*_ is fixed *a priori* (but see the extension by Gruber *et al.* [42]). In more recent work, the dependency structure of the *v*_*i*_ has been estimated from data. The model in Hyvärinen *et al.* [40] in fact contains a parameter matrix that describes the correlations between the *v*_*i*_, and one can estimate these parameters rather straightforwardly [43]. A closely related formalism uses a generative model of the whole covariance structure of **x** [44,45].

Another line of work defines a parametrized pdf that does not have an explicit representation of the variance variables *v*_*i*_ but attempts to model the same kind of dependencies [46,47]. The pdf is typically of the form
6.4
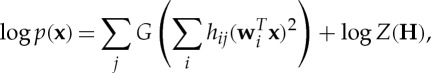
where the **w**_*i*_ are the rows of the separating matrix like in (2.6), the data are whitened, and **W** is constrained orthogonal. (The log-likelihood can be obtained from this formula by just taking the sum over all observed data points **x**(*t*).) What is new here is that instead of taking the nonlinear function *G* of the estimated components 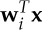 separately, it is taken of the sums of squares. Computing squares is of course intimately related to computing correlations of squares. The matrix **H** describes the dependencies of the linear components 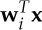. In fact, the nonlinear components 
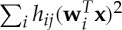
 take the place of the estimated maximally independent components here. We can thus think of this model as a nonlinear version of ICA as well.

The function *Z* in (6.4) is the normalization constant or partition function of the model. What makes estimation of these models challenging is that this function *Z* depends on the parameters *h*_*ij*_ (it is constant only with respect to **x**) and there is no simple formula for it. Computationally simple, general ways of dealing with this problem are considered by Hinton [48], Hyvärinen [49] and Gutmann & Hyvärinen [50], among others, and applied on this model by Osindero *et al.* [46], Köster & Hyvärinen [47] and Gutmann & Hyvärinen [50], respectively.

An alternative approach would be to try to find simple objective functions that are guaranteed to find the right separating matrix in spite of correlations of squares [51,52]. Such methods might be more generally applicable than models that rely on an explicit parametric model of square correlations.

### (c) Dependencies through temporal mixing

In the case of actual time series *x*_*i*_(*t*) and *s*_*i*_(*t*), dependencies between the components (which would usually be called source signals) can obviously have a temporal aspect as well. One starting point is to assume that the innovation processes of the linear components *s*_*i*_(*t*) are independent, whereas the actual time series *s*_*i*_(*t*) are dependent [53]. Using this idea, we can formulate a non-Gaussian state-space model [54,55]. We first model the source signals *s*_*i*_(*t*) using a vector autoregression (VAR) process,
6.5
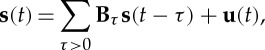
where the **B**_*τ*_ are the autoregressive coefficients, and **u**(*t*) is the innovation process. The innovations *u*_*i*_(*t*) are assumed non-Gaussian and mutually independent, but owing to the temporal mixing by the matrices **B**_*τ*_, the source signals *s*_*i*_(*t*) are not necessarily independent. Then, we model the observed data **x**(*t*) by the conventional mixing model (2.2).

Various methods for estimating such a model have been proposed by Gómez-Herrero *et al.* [54], Zhang & Hyvärinen [55] and Haufe *et al.* [56]. A particularly simple way to estimate the model is to first compute the innovation process of **x**(*t*) by fitting a VAR on it, and then do basic ICA on those innovations, i.e. the residuals [54]. (See also Hyvarinen *et al.* [19] for a related method based on fitting ICA on the residuals of a VAR model.)

Alternatively, we can assume that the components *s*_*i*_(*t*) are independent in a certain frequency band only. If the frequency band is known *a priori*, we can just temporally filter the data to concentrate on that frequency band. In fact, linear temporal filtering does not change the validity of the linear mixing model, nor does it change the mixing matrix. Furthermore, an optimal frequency band can be estimated from the data as well [57].

A different framework of dependent components in time series was proposed by Lahat *et al.* [58], combining the idea of independent subspaces discussed earlier with suitable non-stationarities.

### (d) Further models of dependencies

A model in which the components are linearly correlated (without considering any time structure) was proposed by Sasaki *et al.* [59]. The idea is to consider a generative model similar to the one in (6.2), but with, in some sense, opposite assumptions on the underlying variables: the 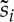 are linearly correlated, while the *v*_*i*_ can be independent (above, it was approximately vice versa). This changes the statistical characteristics because 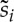 are zero-mean while the *v*_*i*_ are non-negative. In fact, the *s*_*i*_ are then linearly correlated. A topographic kind of dependencies was proposed by Sasaki *et al.* [59].

Very general kinds of dependencies can be modelled by non-parametric models. However, such as all non-parametric models, estimation may require very large amounts of data. A framework modelling dependencies in the form of trees and clusters was proposed by Bach & Jordan [60]. A related approach was proposed by Zoran & Weiss [61].

A recent trend in machine learning is ‘deep learning’, which means learning multi-layer models, where each ‘layer’ is a linear transformation followed by a nonlinear function taken separately of each linear component, like in a neural network [62–64]. In fact, many such models can be considered to be related to ICA: ICA essentially estimates one layer of such a representation. This may lead to the idea that we might just estimate ICA many times, i.e. model the independent components by another ICA, and repeat the procedure. However, this is meaningless because a linear transform of a linear transform is still a linear transform, and thus no new information can be obtained (after the first ICA, any subsequent ICA would just return exactly the same components). Some nonlinearities have to be taken between different layers. The connection between ICA and deep learning models is a very interesting topic for future research.

## 7. Improvements in the estimation of linear decomposition

Finally, we will review methods for more efficient estimation of the basic linear mixing model (2.2) when the components *s*_*i*_ are independent as in the basic model assumptions.

### (a) Independent component analysis using time–frequency decompositions

The basic ICA model assumes that the *s*_*i*_ and *x*_*i*_ are random variables, i.e. they have no time structure. In the basic theory, it is in fact assumed that the observations are independent and identically distribution (i.i.d.), as is typical in statistical theory. However, it is not at all necessary that the components are i.i.d. for ICA to be meaningful. What the i.i.d. assumption means in practice is that any time structure of the data is ignored and what is analysed is simply the marginal distribution of the data over time.

Nevertheless, it is clear that the time structure of the data could be useful for estimating the components. In §6*c*, we already used it to model dependencies between the components, but even in the case of completely independent components, time structure can provide more information. In fact, it is sometimes possible to estimate the ICA model even for Gaussian data, based on the time structure (autocorrelations) alone, as initially pointed out by Tong *et al.* [65] and further developed by Belouchrani *et al.* [34], among others (see ch. 18 of Hyvärinen *et al*. [3] or Yeredor [36] for reviews.) However, such methods based on autocorrelations alone have the serious disadvantage that they only work if the independent components have different autocorrelation structures, i.e. the components must have different statistical properties. This is in stark contrast to basic ICA using non-Gaussianity, which can estimate the model even if all the components have identical statistical properties (essentially, this means equal marginal pdfs).

Thus, it should be useful to develop methods that use both the autocorrelations and non-Gaussianity. In an intuitive sense, such methods would more fully exploit the structure present in the data, leading to smaller estimation errors (e.g. in terms of asymptotic variance). Various combinations of non-Gaussianity and autocorrelations have been proposed. An autoregressive approach was taken in Hyvärinen [66] and Hyvärinen [67]: it is straightforward to construct, for each component, a univariate autoregressive model with non-Gaussian innovations, and formulate the likelihood or some approximation.

Perhaps a more promising recent approach is to use time–frequency decompositions, such as wavelets or short-time Fourier transforms. Pham [68] proposed that we can assume that the distribution of each time–frequency atom (e.g. a wavelet coefficient) of *s*_*i*_(*t*) is Gaussian inside a short time segment. The likelihood of such a Gaussian coefficient is easy to formulate: it is essentially equal to 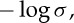 where *σ* is the standard deviation inside the time segment [68]. Note that Gaussianity of the time–frequency atoms does not at all imply the Gaussianity of the whole signals because the variances are typically very different from each other; so we have Gaussian scale mixtures that are known to be non-Gaussian [69]. Related methods with non-Gaussian models for the atoms were developed by Zibulevsky & Pearlmutter [70], and adaptation of the time–frequency decomposition was considered by Pham & Cardoso [71] and Kisilev *et al.* [72]; see Gribonval & Zibulevsky [73] for a review.

A simple practical method for using such a time–frequency decomposition was proposed by Hyvärinen *et al.* [74] (unaware of the earlier work by Pham). Considering the vector of short-time Fourier transforms 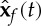 of the observed data vector, we simply take the sum of the log-moduli over each window and component, obtaining
7.1
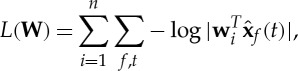
where *t* is the time index, corresponding to the window in which the Fourier transform has been taken, and *f* is the frequency index. Here, a sum of the squares of two Fourier coefficients is implicitly computed by taking the modulus of 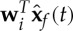, which is complex valued. It can be considered a very rudimentary way of estimating the variance in a time–frequency atom.

This likelihood is to be maximized for orthogonal (or unitary) **W** for whitened data. Comparing this with (2.6), we see that is it remarkably similar in the sense of taking a nonlinear function 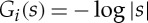 of the estimate of the source, and then summing over both time and frequency. Thus, from an algorithmic viewpoint, the fundamental utility in using (7.1) is that this objective is of the same form as the typical objective functions of a complex-valued ICA model [75], and thus can be performed by algorithms for complex-valued ICA [76]. Taking the time–frequency structure into account is here reduced to a simple *preprocessing* of the data, namely the computation of the time–frequency decomposition.

### (b) Modelling component distributions

In most of the widely used ICA algorithms, the non-quadratic functions *G*_*i*_ are fixed; possibly just their signs are adapted, as is implicitly done in FastICA [77]. From the viewpoint of optimizing the statistical performance of the algorithm, it should be advantageous to learn (estimate) the optimal functions *G*_*i*_. As pointed out already, the optimal *G*_*i*_ has been shown to be the log-pdf of the corresponding independent components [3,4]; so this is essentially a non-parametric problem of estimating the pdfs of the independent components. The problem was analysed on a theoretical level by Chen & Bickel [78], who also proposed a practical method for adapting the *G*_*i*_. Further non-parametric methods were proposed by Vlassis & Motomura [79], Hastie & Tibshirani [80] and Learned-Miller & Fisher [81].

In fact, an ingenious approach to approximating the optimal *G*_*i*_ was proposed much earlier by Pham & Garrat [7], who approximated the derivative of *G*_*i*_ as a linear combination of a set of basis functions. It was shown that the weights needed to best approximate the derivative of *G*_*i*_ can be obtained by a rather simple procedure. It seems that this method has not been widely used mainly because the main software packages for ICA do not implement it, but on a theoretical level, it looks extremely promising.

An alternative approach was proposed by Bach & Jordan [82], in which the fashionable reproducible kernel Hilbert space methods were used to approximate the dependency between two estimated components. The theory was further developed in Gretton *et al.* [83], among others. Another approach using a direct estimate of mutual information was developed by Stögbauer *et al.* [84]. While development of such independence measures is an extremely important topic in statistics, it is not clear what their utility could be in the case of basic ICA, where the problem can be reduced so that we need only *univariate* measures of non-Gaussianity (e.g. differential entropy) as in (2.6), which are simpler to construct than any explicit *multi-variate* (or bivariate) measures of independence.

### (c) Non-negative models

A completely different approach to estimation of a linear mixture model is provided by the idea of using only matrices with non-negative entries in (2.3). This was originally proposed by Paatero & Tapper [85] and Paatero [86] under the heading ‘positive matrix factorization’ in the context of chemometrics, and later popularized by Lee & Seung [87] under the name ‘non-negative matrix factorization’ (NMF).

It is important to understand the meaning of non-negativity here. Of course, many physical measurements, such as mass, length or concentration, are by their very nature non-negative. However, any kind of non-negativity is not sufficient for a successful application of NMF. What seems to be important in practice is that the distribution of the measurements is such that zero has a special meaning, in the sense that the distribution is qualitatively somewhat similar to an exponential distribution. In other words, there should be many observations very close to zero. If you consider measurements of masses that have the average of 1 kg with an approximately Gaussian distribution and a standard deviation of 0.1 kg, it is completely meaningless to use the ‘non-negativity’ of that data. On the other hand, if one computes quantities such as (Fourier) spectra, or histograms, non-negativity may be an important aspect of the data [88] because values in high-dimensional spectra and histograms are often concentrated near zero.

In some cases, such non-negativity constraints in fact enable estimation of the model [89,90] without any assumptions on non-Gaussianity. However, the conditions are not often fullfilled, and in practice, the performance of the methods can be poor. That is why it has been proposed to combine non-negativity with non-Gaussianity, in particular the widespread form of non-Gaussianity called sparseness [91]. Such NMF with sparseness constraints can be seen as a version of the ICA model where the mixing matrix is constrained to be non-negative, and the independent components are modelled by a distribution that is non-negative and sparse (such as the exponential distribution). Furthermore, a similar sparse non-negative Bayesian prior on the elements of the mixing matrix can be assumed. If these assumptions are compatible with the actual structure of the data, estimation of the model can be improved. A closely related ‘non-negative ICA’ approach was proposed by Plumbley [92].

See Plumbley *et al*. [93] for a detailed review, and Cichocki *et al*. [89] for further work including extensions to three-way data.

## 8. Conclusion

It is probably fair to say that in the last 10 years, ICA has become a standard tool in machine learning and signal processing. The generality and potential usefulness of the model were never in question, but in the early days of ICA, there was some doubt about the adequacy of the assumptions of non-Gaussianity and independence. It has been realized that non-Gaussianity is in fact quite widespread in any applications dealing with scientific measurement devices (as opposed to, for example, data in the social and human sciences). On the other hand, independence is now being seen as a useful approximation that is hardly ever strictly true. Fortunately, it does not need to be strictly true because most ICA methods are relatively robust regarding some dependence of the components.

Owing to lack of space, we did not consider applications of ICA here. The applications have become very widespread, and it would hardly be possible to give a comprehensive list anymore. What characterizes the applications of ICA is that they can be found in almost every field of science owing to the generality of the model. On the other hand, each application field is likely to need specific variants of the basic theory. Regarding brain imaging and telecommunications, such specialized literature is already quite extensive. Thus, the future developments in the theory of ICA are likely to be driven by the specific needs of the application fields and may be specific to each such field.
